# Supplementary material for: Behavioral signatures of Y-like neuronal responses in human vision
Source: Sci Rep. 2022 Nov 9;12:19116. doi: 10.1038/s41598-022-23293-8 (PMC9646870; doi:10.1038/s41598-022-23293-8)
Supplement: Supplementary file 1 — Supplementary Legends. [file 41598_2022_23293_MOESM1_ESM.docx]

Title of paper: "Behavioral signatures of Y-like neuronal responses in human vision"

Authors: Ana L. Ramirez, Lowell W. Thompson, Ari Rosenberg, and Curtis L. Baker Jr.

**Supplementary Video S1**

Title: Examples of LM (luminance modulation) and CM (contrast modulation) visual stimuli used for motion direction discrimination task.

Legend:  Examples, as short movie clips, of dynamic visual stimuli used in this study. Left panel, first-order luminance modulation (LM) stimulus at a relatively high spatial frequency (SF), and moderate temporal frequency (TF), with a leftwards direction of motion. Right panel, second-order contrast modulation (CM) stimulus with relatively high carrier SF and TF, with a rightwards direction of envelope motion. Note that for illustration purposes, both of the visual stimuli (LM and CM) have higher contrast than used in the experiments.
